# Development of a breast reconstruction-specific computational model to predict shoulder function in breast cancer survivors

**DOI:** 10.1007/s00520-025-09454-1

**Published:** 2025-04-16

**Authors:** Joshua Pataky, Camille L. Graves, Jared Heitzenrater, Maxime Caru, Meghan E. Vidt

**Affiliations:** 1 Department of Biomedical Engineering, Pennsylvania State University, University Park, PA USA; 2 Department of Public Health Sciences, Penn State College of Medicine, Hershey, PA USA; 3 Department of Pediatrics, Division of Hematology and Oncology, Penn State College of Medicine, Hershey, PA USA; 4 Department of Physical Medicine and Rehabilitation, Penn State College of Medicine, Hershey, PA USA

**Keywords:** Computer simulation, Mastectomy, Breast implants, Breast neoplasms, Pectoralis muscles, Shoulder

## Abstract

**Purpose:**

Mastectomy and reconstruction are treatment components for many breast cancer patients, resulting in long-term shoulder dysfunction. Computational models specific to surgical approach would enable study of underlying mechanisms of shoulder dysfunction, but none exist. Our objectives were as follows: (1) develop and validate models representing lumpectomy, implant-based, and autologous flap-based reconstruction; and (2) determine how muscle contribution to hand acceleration during functional movements differs across models.

**Methods:**

The upper limb model in OpenSim was scaled to force-generating properties and anthropometry of adult females. A 405-cc wrapping surface was placed beneath the pectoralis major muscle path representing subpectoral implant placement. For model validation, shoulder moment was predicted in five postures, with an external load applied equal to mean strength measured from a breast cancer patient cohort. Induced acceleration analysis was used to identify primary muscle contributors to hand acceleration during functional movements.

**Results:**

Following model development, pectoralis major moment arm was reduced in the implant model compared to lumpectomy and flap models. Predicted shoulder moments fell within 1 standard deviation of experimental moments (i.e., external rotation: lumpectomy model, 15.1Nm; implant model, 14.1Nm; flap model, 17.5Nm; experimentally measured, 14.1Nm ± 5.4Nm; 13.0Nm ± 3.6Nm; 15.5Nm ± 5.3Nm, respectively), except horizontal abduction (all groups) and elevation (lumpectomy group), providing validation. Large shoulder muscles, including deltoid, infraspinatus, and subscapularis, were the largest contributors to hand acceleration. Pectoralis major was also identified, possibly relating to post-surgical functional deficits.

**Conclusion:**

This work identified muscle moment arm changes for implant-based reconstruction. These models can be used to predict functional outcomes of differing reconstruction surgeries.

**Supplementary Information:**

The online version contains supplementary material available at 10.1007/s00520-025-09454-1.

## Introduction

Breast cancer is the most common cancer for adult women in the USA, with new diagnoses accounting for 33.2% of cases each year [1]. Despite this high prevalence, 5-year survival rate is over 90% [1]. Surgery is a common treatment, with patients undergoing lumpectomy or mastectomy procedures. Following mastectomy, many patients elect breast reconstruction [2], where reconstruction can be broadly classified into two groups: implants or autologous flaps [3]. Implant-based reconstruction commonly involves elevating the pectoralis major muscle from the chest wall for subpectoral implant placement [4]. Autologous flap reconstruction takes vascularized tissue from other locations in the body to transfer to the breast. Tissue is commonly transferred from the abdomen, including deep inferior epigastric perforators (DIEP) or the transverse rectus abdominus muscles (TRAM). Use of DIEP flaps preserves the patient’s abdominal muscle during tissue harvest, unlike TRAM flaps, which use abdominal muscle to reconstruct the breast. Latissimus dorsi flaps can also be performed, which reflect the latissimus dorsi muscle from the back to the chest for use in breast reconstruction [5]. Ultimately, the reconstruction approach selected for an individual patient is determined by several factors, including patient body habitus and surgeon preference [5]. Previous work reported reduced shoulder function following both implant and flap-based reconstruction, due to surgical trauma to the delto-pectoral region [6–8]. Functional deficits include reduced shoulder strength [9], mobility [10, 11], and altered movement biomechanics [12]. In a cohort of female breast cancer patients undergoing reconstruction, the flap group had reduced shoulder strength compared to the implant group 6 months post-surgery [10]. At 15 months post-surgery, shoulder strength of the flap group improved and no differences were detected between groups [10]. Despite previous work identifying functional deficits following breast reconstruction, the underlying biomechanical mechanisms driving dysfunction are still poorly understood.

The biomechanical mechanisms underpinning shoulder function deficits, like changes to muscle moment arm or muscle force contribution during movement, are difficult to measure in vivo. A muscle’s moment arm represents capacity to produce torque about a joint, and is indicative of that muscle’s leverage [13]. Prolonged contribution from muscles compensating in an injury state can increase secondary injury risk to those muscles [14]. Use of computational musculoskeletal models allows prediction of biomechanical parameters. Models have been previously used to evaluate surgical outcomes, like following joint replacement [15] and tendon transfer [16]. A computational model of the healthy upper limb exists [17], although it does not represent characteristics specific to the breast cancer population. Each surgical approach can alter long-term shoulder function [6–8], but the specific ways function is affected by each surgery are not well known. Developing a model specific to this population will permit evaluation of biomechanical mechanisms of function. For example, a computational model can be used to identify which shoulder muscles provide the largest contribution to hand acceleration during functional movements [18], enabling assessment of how function is impacted across groups. A more thorough understanding of how surgery affects function has the potential to inform surgery prescription to better align with an individual’s post-treatment functional needs and goals.

Our objectives were to (1) develop and validate population-specific computational models representing post-operative outcomes of lumpectomy, implant, and autologous flap-based breast reconstruction and (2) use these models to determine how muscle contribution to hand acceleration during functional movements differs across groups. It was hypothesized that joint torques predicted using inverse dynamics would fall within 1 standard deviation (SD) of measured shoulder strength from a cohort of breast cancer patients for all groups, and that unaffected shoulder muscles with larger volumes, like deltoid and infraspinatus, would have the largest contributions to hand acceleration during functional movements.

## Methods

To develop models representing the female breast cancer population, first, an existing upper limb model was modified. Models were validated using data from patients who underwent breast cancer surgery and reconstruction. Validated reconstruction specific models were then used to perform induced acceleration analyses to understand how muscle contribution to hand acceleration during functional movements differed between breast reconstruction approaches. Data from model development and validation were exported from OpenSim and plotted in Excel (Microsoft Corp., Redmond, WA). Data from induced acceleration analyses were plotted in Matlab (The MathWorks, Inc., Natick, MA). As these modeling analyses result in single outcomes for each surgical approach, no formal statistical analyses could be conducted. Qualitative analyses were performed to determine differences between surgical approaches.

### Model development

**Fig. 1:**
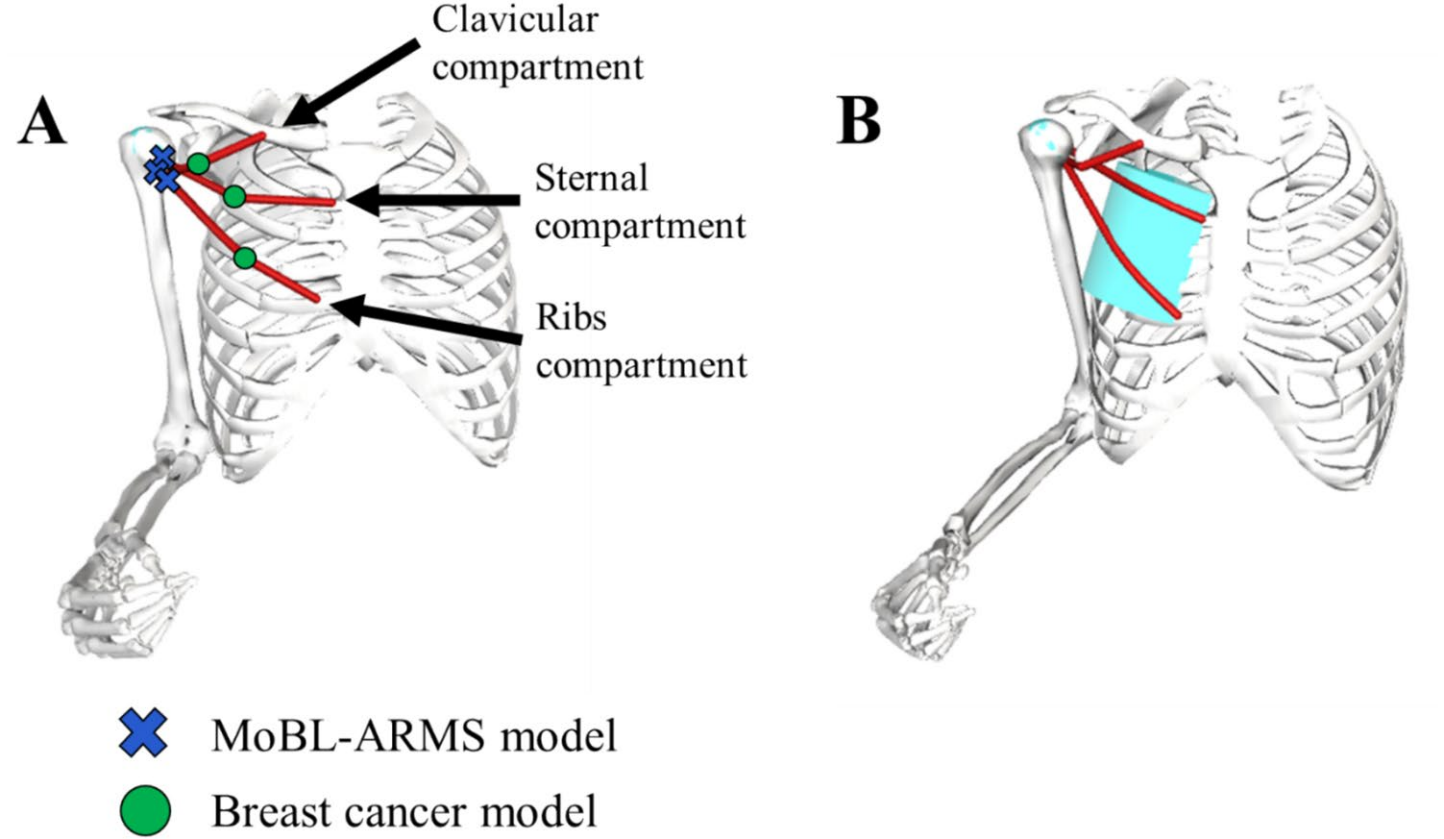
**A** The second muscle path point of the clavicular, sternal, and ribs compartments of pectoralis major were moved from their original locations in the MoBL-ARMS model (blue cross) to better match moment arms reported in literature [22] (green circle). **B** A cylindrical wrapping surface with dimensions matching manufacturer’s specifications for 405-cc breast implant was placed beneath sternal and ribs compartments of pectoralis major muscle paths to represent subpectoral implant placement

The OpenSim platform is an open-source software designed for biomechanical modeling analyses [19]. We started with a baseline model, which was the existing upper limb model (MoBL-ARMS model [17]) in OpenSim v.4.1 [19], that represents the force-generating properties of upper extremity muscles and is scaled to young adult anthropometry [17]. To better represent the female breast cancer population, the force-generating properties of muscle paths in the model were modified to represent those of middle-aged adult females [20]. The model was scaled to 50th percentile adult female anthropometry [21] using OpenSim’s scale tool. It was necessary to ensure the pectoralis major muscle path in the model was anatomically accurate. As subpectoral implant placement requires elevation of pectoralis major muscle from the chest wall, the model muscle path was modified to be anatomically accurate. The three compartments of the pectoralis major muscle path were altered to more closely match moment arms from existing literature [22, 23]. The second muscle path point from the humeral insertion for each of the three pectoralis major muscle compartments (Fig. 1A) was moved to a location that maintained anatomic accuracy of the muscle path, using parameters described in Supplementary Table 1.

Implant and autologous flap reconstruction surgeries replace original breast tissue with an implant (commonly made of silicone) or tissue relocated from the abdomen, respectively, with silicone and abdominal tissue, each of which have different densities than native breast tissue. For implant-based reconstruction, the most common implant volume used is 405 cc [24, 25]. This volume was used to define mass property changes and implant placement during model development. To account for mass property changes within the model, a geometric stadium solid of the torso was created in SolidWorks software (v.2023, Dassault Systèmes SolidWorks Corporation, Waltham, MA), using anthropometric measures of 50th percentile adult females [21]. Native breast tissue is a fatty tissue with density of 0.95 g/mL [26], density of silicone is 1.05 g/mL [27], and density of fatty deep inferior epigastric perforator tissue is 0.84 g/mL [28]. Using the built-in Mass Properties tool in SolidWorks, torso mass, center of mass, and moment of inertia of the torso stadium solid were separately determined for lumpectomy, implant, and flap models (Table 1). Lumpectomy procedures remove only cancerous tissue with approximately 1 cm margin of surrounding tissue, resulting in no detectable change in the center of mass or moment of inertia [29]. To represent the subpectoral implant, a wrapping surface was added to the pectoralis major muscle path in the model (Fig. 1B). A cylindrical wrapping surface matching breast implant manufacturer’s specifications for 405 cc implants [24] was placed beneath sternal and ribs compartments of the pectoralis major muscle paths, consistent with surgical placement. The muscle path points of ribs and sternal pectoralis major compartments between origin and insertion were removed to allow smooth interaction with the wrapping surface (Fig. 1B).

**Table 1:** Parameters implemented in the models to account for different density values of the different tissue or silicone implant used in reconstruction surgery and the implant wrapping surface in the implant model. Projection is defined in centimeters (cm), density in grams per milliliter (g/mL), center of mass defined as the coordinate location in 3-dimensional space (*x y z*) in meters (m) in reference to the origin of the thorax at the suprasternal notch, and moment of inertia (I) in kilogram meters squared (kg·m^2^) about each axis (*I*_*xx*_, *I*_*yy*_, etc.)

**Model**	**Projection (cm)**	**Density (g/mL)**	**Center of mass (m) (*x y z*)**	**Moment of inertia (kg**·**m**^**2**^**)** ***I***_***xx***_*** I***_***xy***_*** I***_***xz***_ ***I***_***yx***_*** I***_***yy***_*** I***_***yz***_ ***I***_***zx***_*** I***_***zy***_*** I***_***zz***_
Lumpectomy	N/A	0.95	(− 0.09262 − 0.16639 0)	0.27165 0.0094687 4.319E- 08 0.0094387 0.1912691 8.3E- 09 4.319E- 08 8.3E- 09 0.2459517
Implant	4.4	1.05	(− 0.09207 − 0.16622 0.00034)	0.272404 0.009904 0.000883 0.009904 0.193346 0.000272 0.000883 0.000272 0.247611
Flap	N/A	0.84	(− 0.09326 − 0.16658 − 0.0004)	0.27077 0.00896 − 0.00103 0.00896 0.188845 − 0.00032 − 0.00103 − 0.00032 0.244015

### Model validation

Each surgery-specific model was validated against measures derived from participants who underwent surgical treatment for breast cancer [30]. The study was performed in accordance with the Declaration of Helsinki and approved by the Pennsylvania State University Institutional Review Board (STUDY00010885); all participants provided voluntary, written informed consent prior to participating in the study. Briefly, participants were divided into three groups based on self-selected surgical treatment and reconstruction approach, including lumpectomy (*n* = 9), mastectomy followed by implant reconstruction (*n* = 9), or mastectomy followed by flap reconstruction (*n* = 7). Shoulder strength was measured using a microFET2 handheld dynamometer (Hoggan Scientific, LLC, Salt Lake City, UT) 3 months after the patient’s terminal surgery (lumpectomy or final reconstruction). Five measures of shoulder strength were taken, including elevation, external rotation, internal rotation, horizontal adduction, and horizontal abduction [31, 32]. Each model was positioned with joint angles matching postures used for data collection. An external load equal to the post-surgical force (strength) measurement was applied to the model at the same location where the handheld dynamometer was placed during data collection (Table 2). The inverse dynamics tool in OpenSim was used to predict joint moment at the shoulder for all thoracohumeral degrees of freedom for each of the five measured postures. Predicted joint moments from inverse dynamics were then compared to post-surgical strength measures.

**Table 2:** Measures of force at the shoulder recorded from a cohort of individuals who underwent lumpectomy, mastectomy followed by implant reconstruction, or mastectomy followed by flap reconstruction in five postures. The mean force measures were input as external loads in Newtons (N) for each model during inverse dynamics simulations for model validation

Group	External rotation (N)	Internal rotation (N)	Horizontal adduction (N)	Horizontal abduction (N)	Elevation (N)
Lumpectomy (*n* = 9)	57.5 ± 21.1	74.8 ± 21.0	82.7 ± 16.5	83.4 ± 24.6	77.1 ± 30.3
Implant (*n* = 9)	53.7 ± 13.1	61.0 ± 22.2	67.9 ± 18.1	64.8 ± 18.7	77.8 ± 31.0
Flap (*n* = 7)	66.6 ± 22.2	76.4 ± 26.0	74.5 ± 25.2	93.6 ± 19.0	95.0 ± 40.6

### Induced acceleration analysis

Once confident that the validated surgery-specific models predicted the breast cancer population, predictive analyses could be performed to better understand functional outcomes. Induced acceleration analyses were performed to determine individual muscle contributions to hand acceleration, as the hand is the most relevant segment to evaluate upper limb functional task performance. Prior to analyses, static kinematic time histories matching the postures used to experimentally measure shoulder strength were developed. Three motions were developed, including external-internal rotation, elevation in the scapular plane, and horizontal abduction–adduction. External-internal rotation started in neutral posture, externally rotated to 45°, paused for a moment, then internally rotated to 30° (Supplementary Fig. 1 A). Elevation in the scapular plane started at 45° of abduction, abducted to 90°, paused for a moment, then adducted back to the starting position (Supplementary Fig. 1B). Horizontal abduction–adduction started with the model supine and humerus flexed to 90°, abducted to 90°, and internally rotated to 90°. The humerus was then horizontally abducted 45°, then adducted 30° past the starting position (Supplementary Fig. 1 C). Each motion, along with the three models, were used as inputs to OpenSim’s computed muscle control algorithm [33]. Briefly, the computed muscle control algorithm was used to calculate predicted muscle activations for each muscle path actuator that was needed to track the input kinematic time history. Muscle activations and joint angles from computed muscle control were then used as inputs to the induced acceleration analysis. The induced acceleration tool in OpenSim sets the force of all muscles but one to zero, and resulting accelerations of the hand are calculated; this is repeated iteratively for all 50 muscle paths in the model [18]. Accelerations contributed by each muscle path were evaluated along each of the anterior–posterior, superior-inferior, and lateral-medial axes of the thorax segment.

## Results

### Model development and validation

**Fig. 2:**
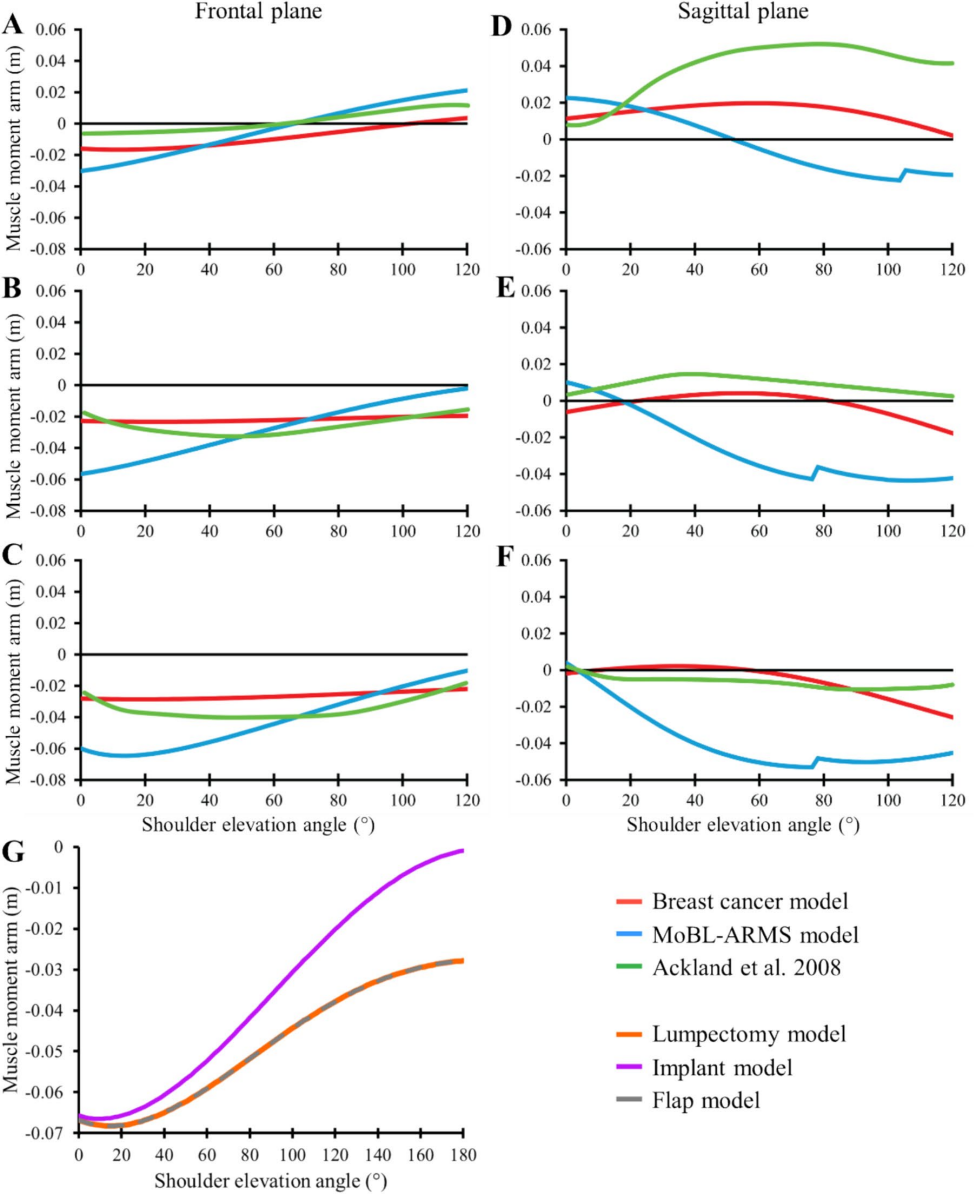
Pectoralis major moment arm changes during model development. Moment arms of pectoralis major muscle compartments from literature [22] (green), the existing MoBL-ARMS model (blue), and the developed breast cancer model (red) to more closely match literature moment arms. Clavicular (**A**, **D**), sternal (**B**, **E**), and ribs (**C**, **F**) compartments of pectoralis major were compared with the humerus at 90° shoulder elevation in the frontal (**A**, **B**, **C**) and sagittal (**D**, **E**, **F**) planes. **G** Pectoralis major moment arm for the lumpectomy (red), implant (blue), and flap (green) models. There are no differences between lumpectomy and flap model moment arms, as muscle paths were not changed during development. The moment arm of the implant model is reduced compared to the lumpectomy and flap models. Figure data was exported from OpenSim and plotted in Excel (Microsoft Corp., Redmond, WA)

Following adjustment of pectoralis major muscle path points, the moment arms of each pectoralis major compartment more closely matched empirical measurements in literature (Fig. 2) [22, 23]. After implementing the wrapping surface to represent subpectoral implant placement, the moment arm of the summed muscle paths was reduced in magnitude, more closely matching literature (Fig. 2). There were no differences in summed pectoralis major moment arm between lumpectomy and flap models.

**Fig. 3:**
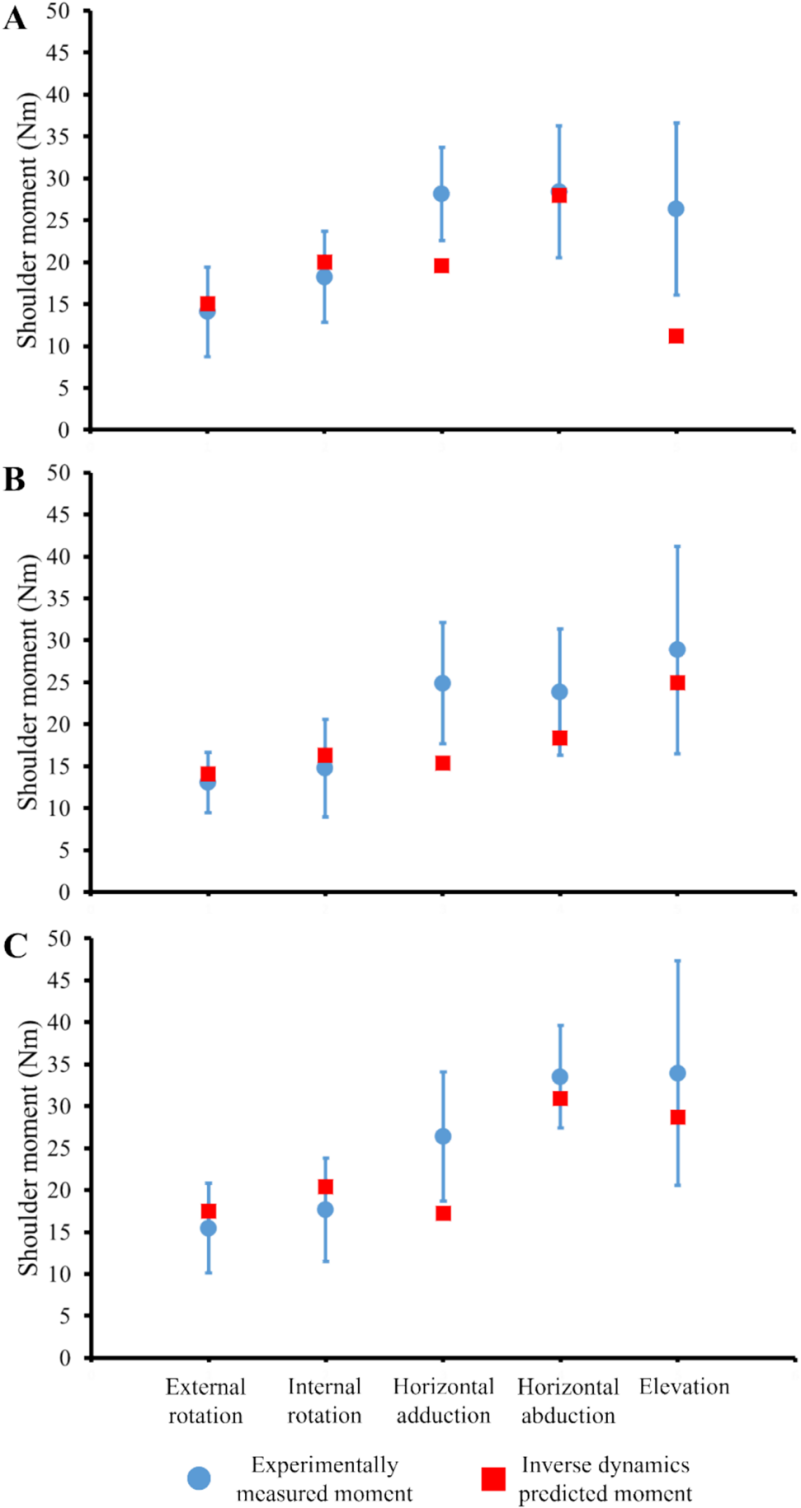
Predicted joint moment (red square) for the **A** lumpectomy, **B** implant, and **C** flap models for each of the five postures compared to the mean (blue circle) and 1 standard deviation (error bar) of measured shoulder moments from a cohort of breast cancer patients. Figure data was exported from OpenSim and plotted in Excel (Microsoft Corp., Redmond, WA)

Joint moments predicted by inverse dynamics matched experimentally measured shoulder moments (strength) for all models and postures (Fig. 3), which indicated validation of developed models. For the lumpectomy model, predicted joint moments were within 1SD of the mean for the primary degree of freedom for all postures except horizontal adduction and elevation, with 15.1 Nm for external rotation (experimentally measured, 14.1 Nm ± 5.4 Nm), 20.0 Nm for internal rotation (experimentally measured, 18.3 Nm ± 5.4 Nm), and 28 Nm for horizontal abduction (experimentally measured, 28.4 Nm ± 7.9 Nm). Predicted moments for horizontal adduction was 19.6 Nm (experimentally measured, 28.1 Nm ± 5.6 Nm) and elevation was 11.2 Nm (experimentally measured, 26.3 Nm ± 10.2 Nm), falling outside 1SD but within 2SD of the experimentally measured mean. Joint moments predicted using the implant model also fell within 1SD of the mean for all postures except horizontal adduction, with 14.1 Nm for external rotation (experimentally measured, 13.0 Nm ± 3.6 Nm), 16.3 Nm for internal rotation (experimentally measured, 14.8 Nm ± 5.8 Nm), 18.3 Nm for horizontal abduction (experimentally measured, 23.9 Nm ± 7.5 Nm), and 24.9 Nm for elevation (experimentally measured, 28.8 Nm ± 12.3 Nm). For horizontal adduction, predicted joint moment was 15.4 Nm (experimentally measured, 24.9 Nm ± 7.2 Nm), which was outside 1SD but within 2SD of the experimentally measured mean. For the flap model, predicted joint moments also matched measured shoulder strength for all postures except horizontal adduction, with 17.5 Nm for external rotation (experimentally measured, 15.5 Nm ± 5.3 Nm), 20.4 Nm for internal rotation (experimentally measured, 17.7 Nm ± 6.1 Nm), 30.9 Nm for horizontal abduction (experimentally measured, 33.5 Nm ± 6.1 Nm), and 28.7 Nm for elevation (experimentally measured, 33.9 Nm ± 13.4 Nm). For horizontal abduction, predicted joint moment was 17.2 Nm (experimentally measured, 26.4 Nm ± 7.7 Nm), which was outside 1SD but within 2SD of the experimentally measured mean.

### Induced acceleration analysis

**Fig. 4:**
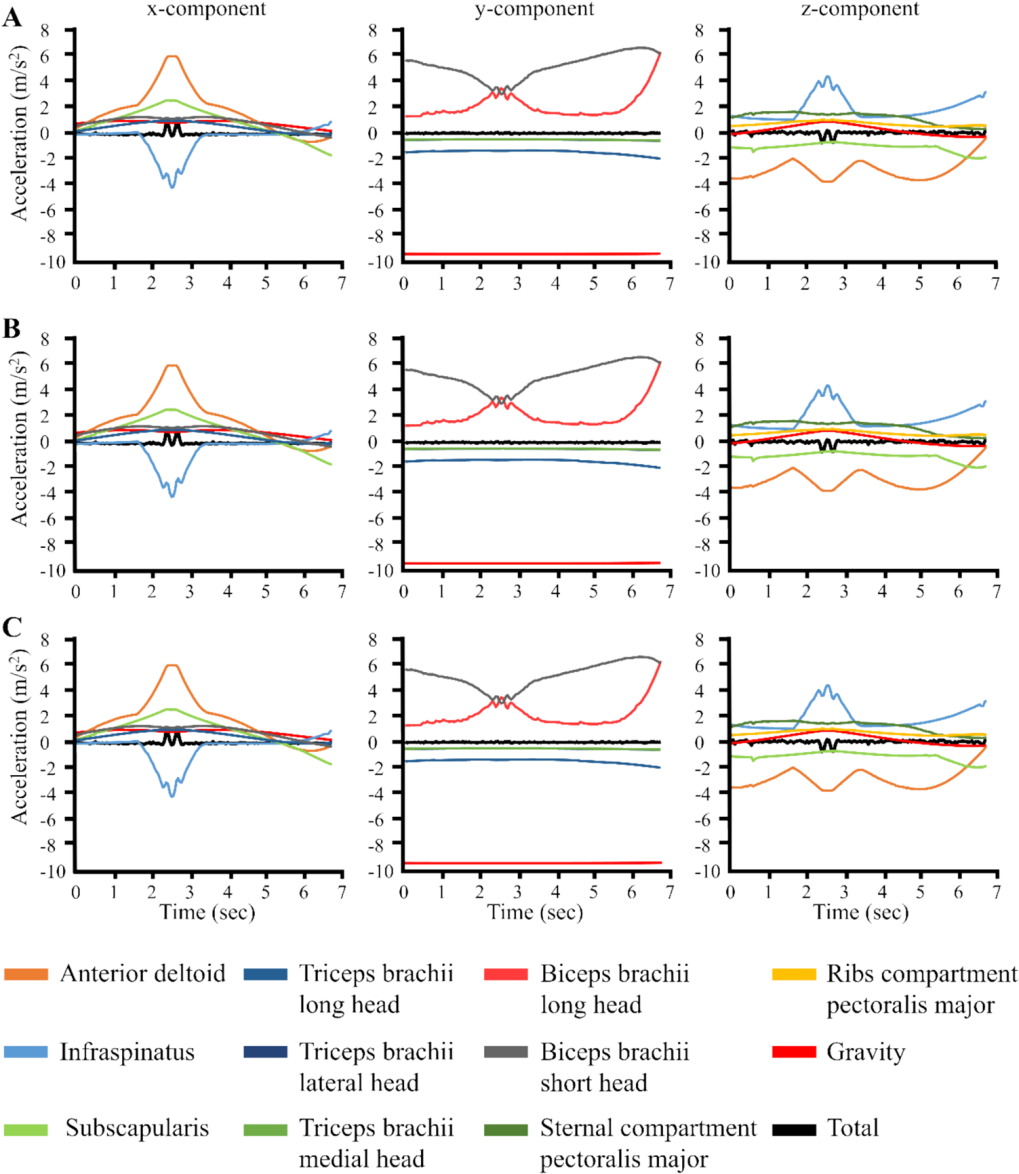
Muscles with the largest contribution towards hand acceleration determined using induced acceleration analysis for the **A** lumpectomy, **B** implant, and **C** flap model during the external-internal rotation motion. Accelerations were evaluated along the anterior–posterior (*x*), superior-inferior (*y*), and lateral-medial (*z*) axes of the thorax. Data was plotted in Matlab (The MathWorks, Inc., Natick, MA)

Results of induced acceleration analysis revealed the five muscle paths with greatest contributions to hand acceleration along each axis in the thorax coordinate system (Fig. 4). For the external-internal rotation motion using lumpectomy (Fig. 4A), implant (Fig. 4B), and flap (Fig. 4C) models, muscles with the greatest contributions along the anterior–posterior axis were anterior deltoid, infraspinatus, subscapularis, triceps brachii long head, and biceps brachii short head. Along the superior-inferior axis, greatest contributors to hand acceleration were triceps brachii long head, triceps brachii lateral head, triceps brachii medial head, biceps brachii long head, and biceps brachii short head. The greatest contributors along the lateral-medial axis were anterior deltoid, infraspinatus, subscapularis, sternal compartment of pectoralis major, and ribs compartment of pectoralis major. For elevation motion in the scapular plane (Supplementary Fig. 2), the largest contributors to hand acceleration along the anterior–posterior axis for all models were infraspinatus, teres minor, sternal and rib compartments of pectoralis major, and long head of biceps brachii. Along the superior-inferior axis, the largest contributors were middle deltoid, supraspinatus, infraspinatus, biceps brachii long head, and biceps brachii short head. Along the lateral-medial axis, the largest contributors were teres major, sternal and ribs compartments of pectoralis major, and thoracic and lumbar compartments of latissimus dorsi. For the horizontal abduction–adduction motion (Supplementary Fig. 3), the largest contributors to acceleration along all axes for all models were infraspinatus, subscapularis, triceps brachii long head, triceps brachii lateral head, triceps brachii medial head, biceps brachii long head, biceps brachii short head, and sternal compartment of pectoralis major. Small fluctuations in muscle contribution to hand acceleration occurred at the task midpoint due to the transition from the dynamic task to a static posture (pausing for a moment) before resuming the dynamic task.

## Discussion

This study developed and validated computational models representing the surgical breast cancer population, including models for lumpectomy, breast reconstruction with implants, and breast reconstruction with autologous flaps. Subpectoral implant placement decreased pectoralis major moment arm across shoulder elevation. Predicted shoulder moments matched measures recorded from a cohort of breast cancer patients after surgery. Induced acceleration analysis was performed using these models to determine the muscle paths with greatest contribution to hand acceleration during functional movements.

The model representing flap-based reconstruction surgery did not alter moment arms of muscles crossing the shoulder, as DIEP flaps do not involve sub-pectoral placement of grafted tissue [34]. For the implant-based model, pectoralis major muscle moment arm was reduced in magnitude from implant placement beneath sternal and ribs muscle compartments. Muscles with larger moment arms often function as prime movers, due to greater mechanical advantage and potential to contribute to joint torque [35, 36]. Thus, moment arm reductions for this large muscle reduce mechanical advantage for joint movement [35, 36]. With its smaller moment arm, pectoralis major can still function as a joint stabilizer, but with reduced shoulder torque production [35, 36]. This finding is consistent with a previous gait study that showed compromised Achilles tendon moment arm reduced ankle torque and limited walking ability [37]. Reductions in moment arm seen here could contribute to the reduced shoulder strength and function reported in literature [9–12].

Model validation with inverse dynamics simulations showed shoulder moments for most postures fell within 1SD of the mean of experimentally measured joint moments. Simulations examined external-internal rotation, horizontal abduction, and flexion-elevation tasks, each of which uses a large proportion of the upper limb workspace [38]. Horizontal adduction moment was the only posture consistently smaller than the mean experimentally measured moment for all models. This motion is a combination of shoulder degrees of freedom, however for this study, shoulder moment was evaluated for an isolated degree of freedom (elevation plane) during model validation. Additionally, muscles, including trapezius and serratus anterior, contribute to horizontal abduction, but the current model does not include these representations. More work is needed to include these muscle paths in the model. Small differences between predicted and experimentally measured moments in other postures could be due to model scaling to 50th percentile adult female anthropometry, while experimental moments were collected using arm segment lengths and forces from individual patients. The models developed here are group-specific, with predicted moments falling within 1SD, indicating successful model validation. The developed models represent 50th percentile adult female anthropometry, while patients tested ranged from 1st to 98th percentile in height for adult females [21]. Scaling the model to individual-specific anthropometry with individualized forces could further minimize the differences seen here. Nevertheless, these population-specific models can be used to predict shoulder moments for a wide range of postures and tasks without extensive in vivo testing. The ability to use validated, population-specific models to study complex functional upper extremity movements enables more detailed predictions of biomechanical parameters, like shoulder moment, enhancing our understanding of specific functional changes following breast surgery and reconstruction.

Outcomes of induced acceleration analyses revealed that infraspinatus, subscapularis, and anterior deltoid muscles were the largest contributors to hand acceleration across models and functional tasks. These muscles are larger muscles by volume in the shoulder complex [39], confirming the hypothesis that muscles with larger volume would be among the largest contributors to hand acceleration during functional tasks. Triceps brachii long head, triceps brachii lateral head, triceps brachii medial head, bicep brachii long head, and biceps brachii short head muscles were large contributors toward hand acceleration along the superior-inferior axis of the torso. Both sternal and ribs compartments of pectoralis major muscle also had large contributions to hand acceleration. Despite pectoralis major muscle compartments being relatively smaller in volume, they are primary shoulder movers that are being modified following reconstruction surgery. The current study’s finding that pectoralis major is a primary contributor to functional movement could help explain why patients who have surgery that disrupts this muscle, such as implant reconstruction, report post-surgical functional deficits [9, 10]. Further analysis of induced accelerations during additional functional movements could help delineate specific changes in muscle contribution to motion that occur following reconstruction surgery [40]. By targeting the muscles with the largest contribution to hand acceleration identified in this study with rehabilitation and strengthening programs, muscle strength and coordination can increase, which has potential to reduce shoulder dysfunction and offset long-term mobility losses [41, 42]. The outcomes and models described here can also be used to optimize surgical methods to help preserve muscles, such as pectoralis major, following surgery, which are vital for upper extremity function [43].

This work has some limitations. For the implant-based model, the most common implant size of 405 cc was represented, although implants range in size from 150 to 750 cc [24, 25]. Future work investigating the effect of implant size could show that smaller implant sizes may have less effects on biomechanical parameters, like moment arm, while larger implant sizes may result in a more pronounced effect. For the flap-based model, tissue density changes were based on DIEP tissue flaps, but other autologous tissues, such as the latissimus dorsi muscle, are used clinically. This reconstruction approach could have implications on function by reducing muscle volume, in addition to the impacts on fatigue previously reported following surgery [6, 44]. Future work will further develop the models presented here to encompass the range of breast sizes and other types of tissues used in reconstruction surgery. After validation, models fell within 1SD of experimental strength measures for most postures, but further model refinement could result in closer predictions. The effects of other cancer treatments, such as chemotherapy or radiation, are not directly represented in these models, which could include reduced muscle volume or increased muscle stiffness [45]. While these effects were indirectly included in the experimental measurements used for validation, some participants from whom strength measures were obtained did receive these other treatments in conjunction with surgery. More work is needed to fully understand the biomechanical implications of adjuvant treatments on muscle size and force-generating parameters so these effects can be modeled.

These models can be used to predict functional outcomes following breast cancer surgery and reconstruction, and optimize surgical methods to preserve muscles that primarily contribute to functional movements. New strategies to prevent functional deficits and reduce long-term burden for breast cancer survivors are needed, and the models developed and validated here can be a useful tool to help identify mechanisms driving shoulder dysfunction. There is a high potential for future clinical translation to provide further information in the shared decision-making pathway between providers and patients.

## Conclusion

This work developed computational models specific to implant and autologous flap breast reconstruction surgery. Models were used to determine that deltoid, infraspinatus, subscapularis, triceps brachii, and biceps brachii muscles are the largest contributors to hand acceleration during common functional movements, highlighting their primary role in shoulder function. Sternal and ribs compartments were also major contributors to hand acceleration; as these muscle paths are commonly disrupted during surgery, particularly for subpectoral implant placement, this could account for reduced shoulder function reported by post-surgery. The models developed here can be used to better predict functional outcomes of differing reconstruction surgeries and be tools to develop novel treatment and rehabilitation strategies to offset functional burdens of disease.

## Supplementary Information

Below is the link to the electronic supplementary material.

Supplementary file1 (DOCX 887 KB)

## Data Availability

No datasets were generated or analysed during the current study.
