# Supplementary material for: Development of a breast reconstruction-specific computational model to predict shoulder function in breast cancer survivors
Source: Support Care Cancer. 2025 Apr 16;33(5):390. doi: 10.1007/s00520-025-09454-1 (PMC12003432; doi:10.1007/s00520-025-09454-1)
Supplement: Supplementary file 1 — Supplementary file1 (DOCX 887 KB) [file 520_2025_9454_MOESM1_ESM.docx]

**Supplemental**

**Table**1**:** The second path point in each of the pectoralis major muscle paths was altered to better match the muscle moment arms in previous cadaveric studies [22]. Coordinate locations (x y z) are described in meters (m) in reference to the origin of the thorax, located at the suprasternal notch.

| **Pectoralis major muscle compartment** | **Breast cancer model**  **(m) (x y z)** |
| --- | --- |
| Clavicular pectoralis major | (0.003, -0.003, 0.131) |
| Sternal pectoralis major | (0.034, -0.044, 0.108) |
| Ribs pectoralis major | (0.059, -0.094, 0.1) |


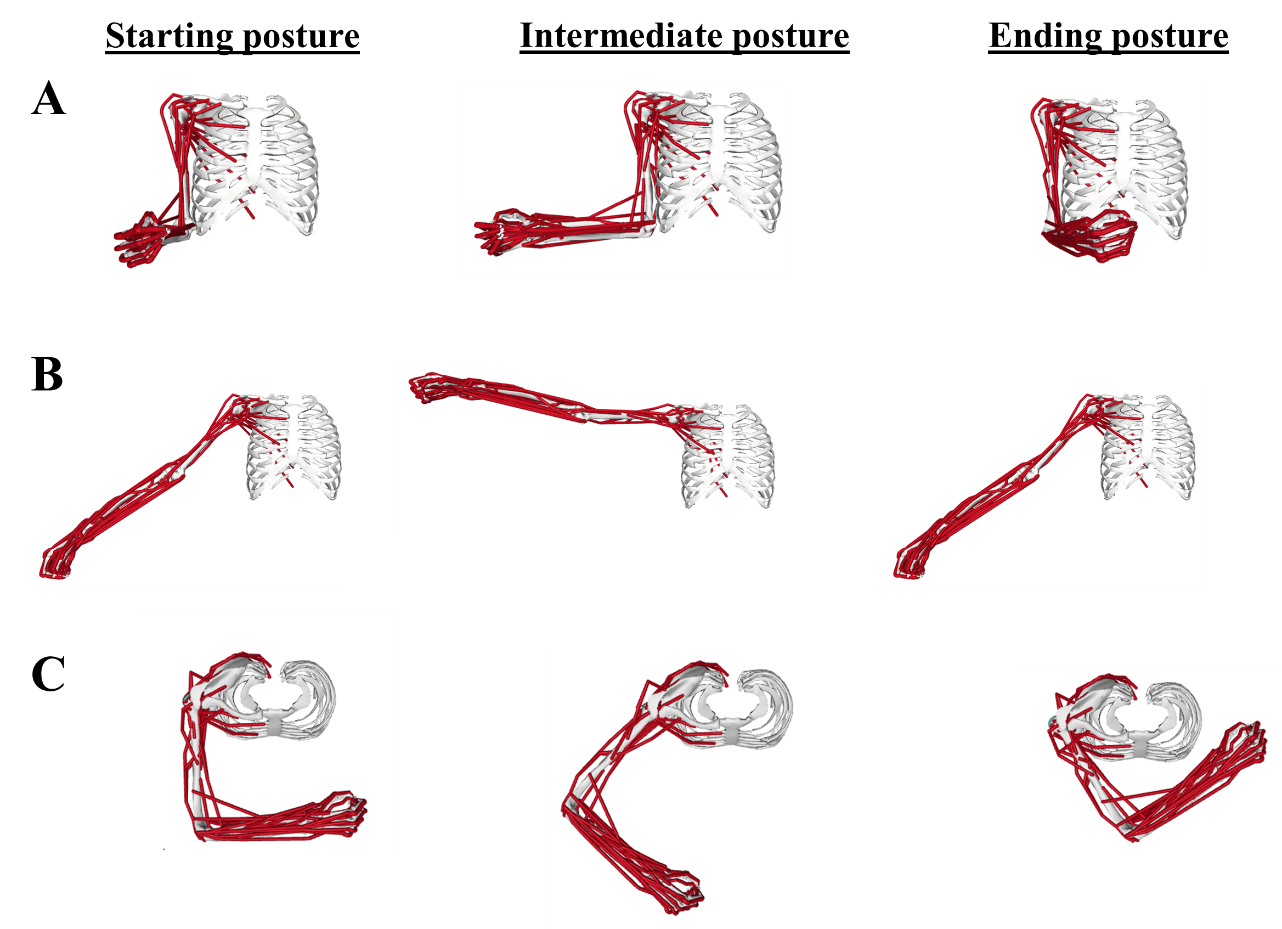


**Figure 1:** Three motions that use a broad range of the upper limb workspace to predict the acceleration of the hand using induced acceleration analysis. (A) External-internal rotation started in neutral posture, externally rotated to 45°, then internally rotated to 30°. (B) Elevation in the scapular plane started at 45° of abduction, abducted to 90°, and then adducted back to the starting position. (C) Horizontal abduction-adduction started with the model supine, and the humerus flexed to 90°, abducted to 90°, and internally rotated to 90°. The humerus was then horizontally abducted 45°, then adducted 30° past the starting position.


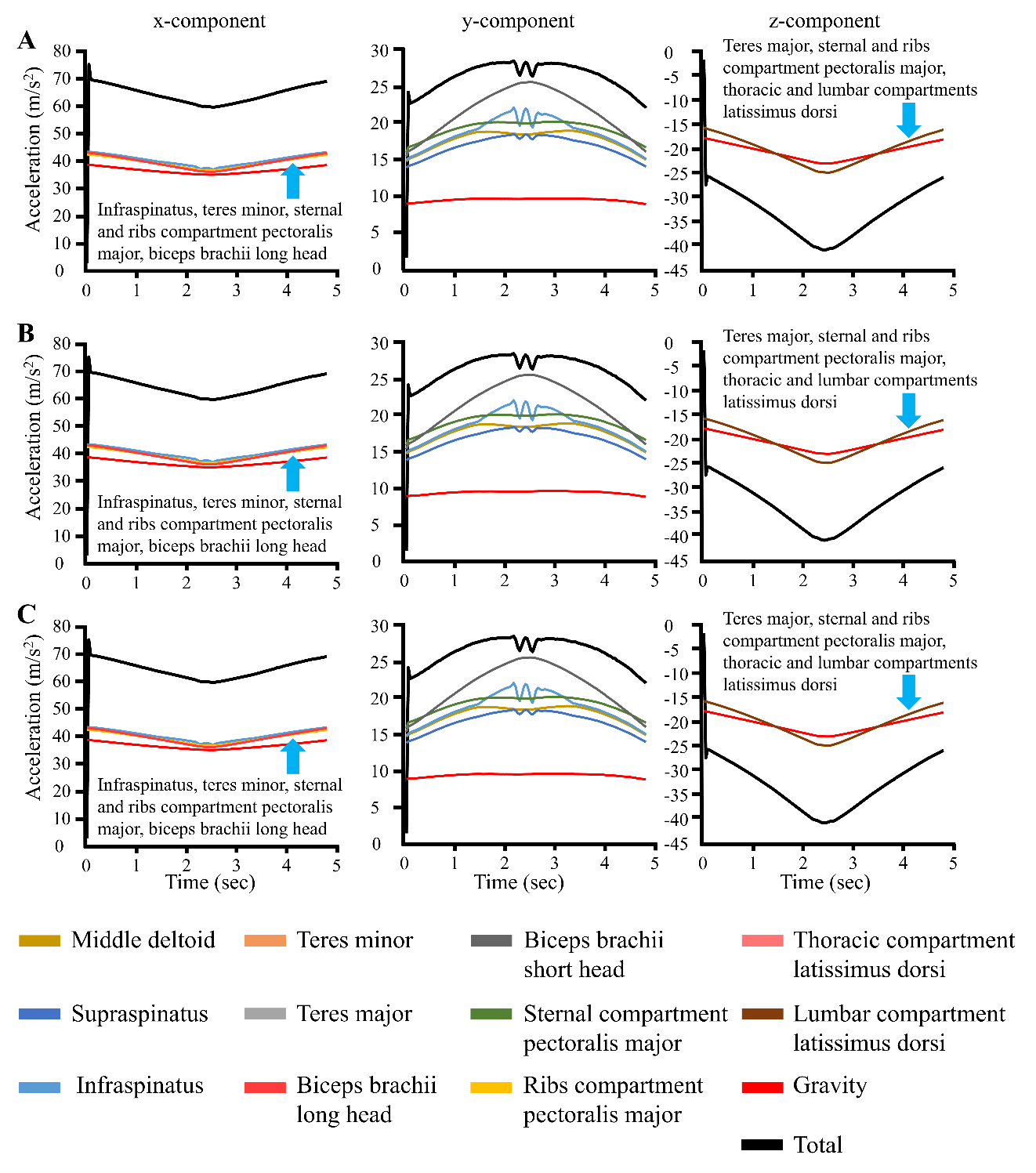


**Figure 2:** Muscles with the largest contribution towards hand acceleration determined using induced acceleration analysis for the (A) lumpectomy, (B) implant, and (C) flap model during the elevation in the scapular plane motion. Accelerations were evaluated along the anterior-posterior (x), superior-inferior (y), and lateral-medial (z) axes of the thorax. Data was plotted in Matlab (The MathWorks, Inc., Natick, MA).


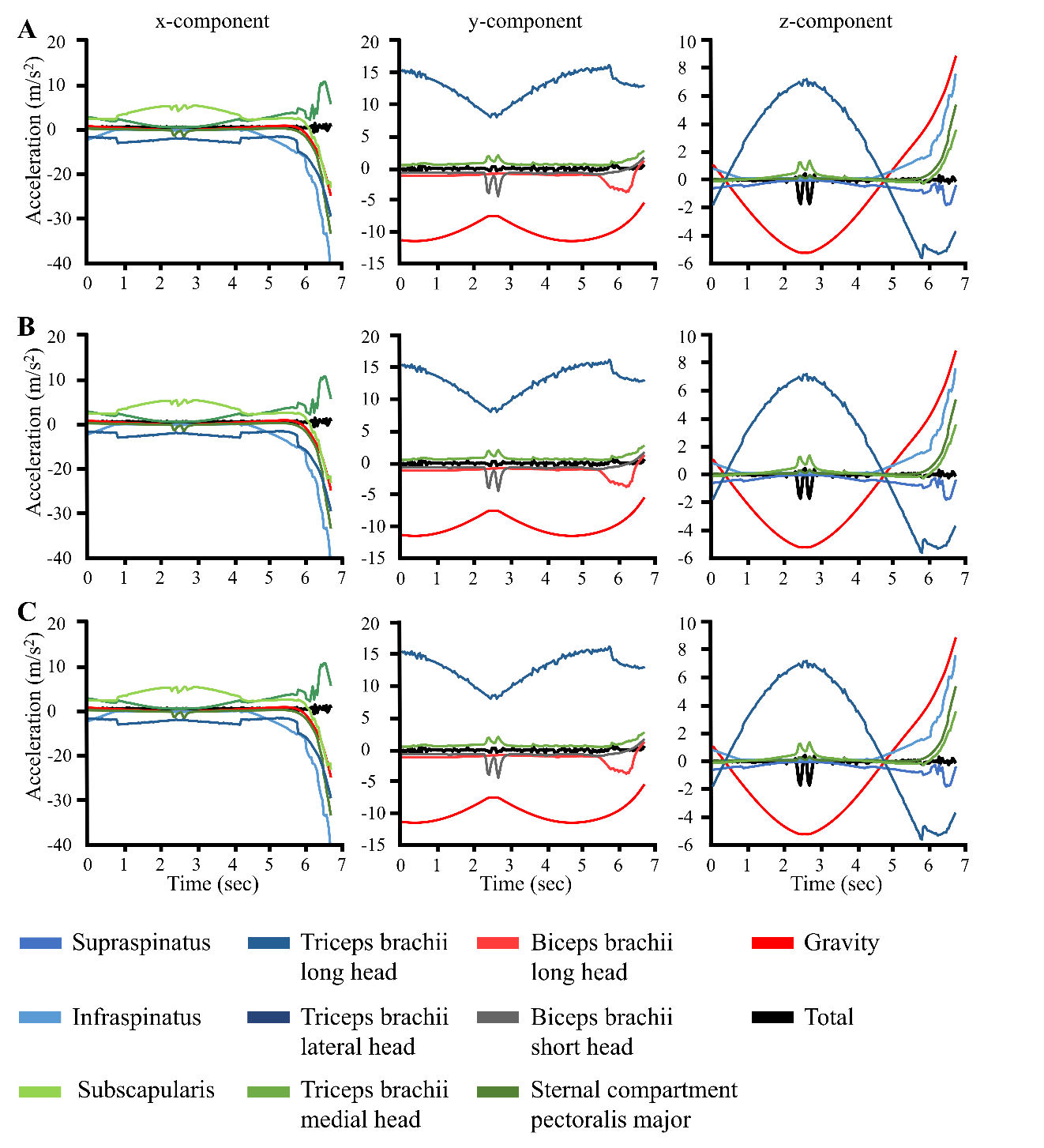


**Figure 3:** Muscles with the largest contribution towards hand acceleration determined using induced acceleration analysis for the (A) lumpectomy, (B) implant, and (C) flap model during the horizontal abduction-adduction motion. Accelerations were evaluated along the anterior-posterior (x), superior-inferior (y), and lateral-medial (z) axes of the thorax. Data was plotted in Matlab (The MathWorks, Inc., Natick, MA).
